# Isolation, Characterization, and Inactivation of *Stenotrophomonas maltophilia* From Leafy Green Vegetables and Urban Agriculture Systems

**DOI:** 10.3389/fmicb.2019.02718

**Published:** 2019-11-29

**Authors:** Dan Li, Chun Hong Wong, Mei Fang Seet, Nicole Kuan

**Affiliations:** Department of Food Science & Technology, Faculty of Science, National University of Singapore, Singapore, Singapore

**Keywords:** *Stenotrophomonas maltophilia*, opportunistic pathogen, leafy green vegetables, food safety, urban agriculture

## Abstract

*Stenotrophomonas maltophilia* is an emerging opportunistic pathogen that, on the one hand, causes severe nosocomial infection in immunocompromised populations with a high mortality rate and, on the other hand, is present ubiquitously in the environment. This study, for the first time to the best of our knowledge, isolated and characterized *S. maltophilia* from leafy green vegetables produced by hydroponic farms and from a hydroponic farming facility in Singapore. Eleven *S. maltophilia* isolates were obtained from three types of leafy green vegetables (sweet basil, kale, and parsley) and from the nutrient solution used by a hydroponic farm. The antimicrobial resistance (AMR), biofilm-forming ability, and resistance to UV and quaternary ammonium compound (QAC) treatments were investigated, as was the fate of *S. maltophilia* in a simulated leafy green vegetable environment during a storage period of 6 days at different temperatures. The results showed that high population levels of *S. maltophilia* could be reached on leafy green vegetables, especially after being stored at abused temperatures (>8-log CFU/ml in basil juice after 6 days storage at 20°C) and on hydroponic farming facilities, probably due to biofilm formation (8 to 9-log CFU/well in biofilms). At 4°C, *S. maltophilia* was able to survive, but no growth was observed during storage in either bacteria culture media or basil juice for a period of 6 days. UV treatment, which induced substantial reductions in *S. maltophilia* in both single-species and dual-species biofilms mixed with *Salmonella enterica* serovar Typhimurium reference strain (ATCC 14028) or self-isolated *Pseudomonas fluorescens* (>4-log reductions by 250 mJ/cm^2^ UV), is recommended for employment by hydroponic farms to treat their nutrient solutions and farming facilities so as to enhance microbial safety.

## Introduction

Opportunistic pathogens typically do not cause disease in healthy individuals. They are only able to exhibit virulence when there is a perturbation to the host immune system, such as pre-existing or prior diseases and infections, immunosuppressive medications, and aging (Brown et al., 2012). In recent years, the demographic shift toward an aging population as well as higher proportions of vulnerable individuals facing immunosuppressive diseases and treatments indicates that an increasing proportion of people are at risk of infections caused by opportunistic pathogens (Newell et al., 2010). *Stenotrophomonas maltophilia* is an important opportunistic pathogen that has been reported to be associated with a wide variety of infections most commonly related (but not limited) to the respiratory tract, such as pneumonia and acute exacerbations of chronic obstructive pulmonary disease (Looney et al., 2009; Adegoke et al., 2017). In particular, the mortality rate was revealed by a meta-analysis of 13 studies to be up to 37.5% across the different types of *S. maltophilia* infections (Falagas et al., 2009).

The transmission of *S. maltophilia* has been mainly attributed to nosocomial routes because, in health-care settings (e.g., wards, outpatient hemodialysis units, or same-day surgery), the number of immunocompromised individuals is high (Berg et al., 2014). However, on the other hand, *S. maltophilia* is known to be present ubiquitously in aqueous environments, plant rhizospheres, etc. (Brooke, 2012). In fact, it is also used as a biocontrol or stress-protecting agent for crops in sustainable agriculture as well as in bioremediation strategies (Berg and Martinez, 2015). Since it is still impossible to distinguish beneficial from harmful *S. maltophilia* strains (Berg and Martinez, 2015), the possibility of foodborne *S. maltophilia* infection cannot be ruled out.

Leafy green vegetables are widely recognized as healthy foods, and their consumption has been growing enormously worldwide in recent years. However, leafy green vegetables, naturally harboring a high surface population of microbes, are also a potential vehicle of microbial pathogen transmission (Berg et al., 2014). Nowadays, due to sustainability reasons, the production of leafy green vegetables by urban agricultural systems, especially by hydroponic systems, is becoming increasingly popular worldwide. With the use of urban agricultural systems, many risk factors can be completely or partially avoided, such as wildlife, livestock, flooding, etc. However, new possible microbial risks can be introduced due to, for instance, closed-loop irrigation systems.

In this study, *S. maltophilia* were isolated from multiple hydroponically produced leafy green vegetables as well as the nutrient solution used at a hydroponic farm. The characteristics of the *S. maltophilia* isolates, including AMR, biofilm-forming ability, and resistance to UV and quaternary ammonium compound (QAC) treatments were investigated, as was the fate of the bacteria during a storage period for 6 days at different temperatures.

## Materials and Methods

### Isolation of *S. maltophilia* From Leafy Green Vegetables and Hydroponic Farming Water

Sweet basil, parsley, and kale (produced by local hydroponic farms, according to their packaging) were purchased from a local supermarket (stored in chiller cabinets) and transported to the laboratory in a cooler bag. Two batches (500 g per batch) of sweet basil were purchased on September 26, 2018 and October 8, 2018, and one batch each (500 g per batch) of parsley and kale were purchased on January 8, 2019. Farm water (a nutrient solution that has direct contact with the roots of plants) was sampled from a running hydroponic facility at a local farm on January 22, 2019, collected in three sterile 15 mL screw-capped tubes, and transported to the laboratory in an ice box. The leafy greens and farm water samples were stored in a refrigerator at approximately 4°C prior to the start of the experiment within the day of sampling.

Five grams of each type of leafy green vegetable was stomached with 45 mL of Buffered Peptone Water (BPW) (Oxoid, Hampshire, United Kingdom) for 2 min using a stomacher (BagMixer^®^ 400 CC^®^, Interscience, Paris, France). Serial dilution of the stomached solutions and farm water samples (pooled from the three water samples) were inoculated onto VIA (*Stenotrophomonas* Selective Agar with vancomycin, imipenem, and amphotericin B; HiMedia Laboratories Pvt. Ltd., Mumbai, India) plates and incubated at 30°C for 48 h. Presumptive *S. maltophilia* colonies (dark green, circular, non-mucoid) were picked out, streaked on new VIA agar plates, and incubated at 30°C for another 48 h. This was to make a secondary purification of the selected colonies of *S. maltophilia* on the VIA agar plates: a dark, translucent, green smear with small and circular single colonies.

### Confirmation of *S. maltophilia* by API 20 NE

For each presumptive isolate of *S. maltophilia*, a loopful of bacteria was streaked onto a Tryptone Soya Agar (TSA) (Oxoid, Hampshire, United Kingdom) plate and incubated at 30°C for 24 h. Colonies on TSA plates were picked and transferred to 5 mL of 0.85% physiological saline solution (Goodrich Chemical Enterprise, Singapore) until the bacterial suspension reached a turbidity that matched the 0.5 McFarland standard. The 0.5 McFarland standard was prepared by adding 0.05 mL of 1.175% barium chloride dihydrate solution (Sigma-Aldrich, MO, United States) to 9.95 mL of 1% sulfuric acid solution (Merck, New Jersey, United States). The bacterial suspensions were inoculated into the API 20NE strips (bioMérieux, Marcy-l’Étoile, France) following the instructions from the manufacturer. The strips were incubated at 30°C for 24 h prior to the reading of the results. A seven-digit profile number was generated by comparing the phenotyping results from the strip with the reading table supplied in the kit, and a significant taxa percentage was generated based on the seven-digit profile number by the apiweb^TM^ database.

### 16S rDNA Gene Sequencing Analysis

Bacterial DNA was extracted with the use of a GeneJet Genomic DNA Extraction Kit (ThermoScientific, United States) according to the manufacturer’s instructions. The 16S rDNA was amplified using Taq polymerase (Promega, United Kingdom) with 0.2 μM of 27F and 1492R primers in a 50 μl reaction (27F: AGAGTTTGATCMTGGCTCAG, 1492R: TACCTTGTTACGACTT). The optimal thermocycling conditions were as follows: 95°C hold for 15 min, 35 cycles of 94°C for 1 min, 52°C for 1.5 min and 70°C for 1.5 min, followed by 5 min at 70°C. PCR products were visualized by electrophoresis using a 1% agarose gel and staining with FloroSafe DNA stain (1st Base, Singapore). The amplified DNA was purified using the GeneJet Gel Purification Kit (ThermoScientific, United States) according to the manufacturer’s instructions. The concentration of the purified DNA products was measured by BioDrop μLITE+ (BioDrop, United Kingdom). TA cloning was performed using pGEM^®^-T Easy Vector Systems (Promega, United States) according to the manufacturer’s instructions. Transformed JM109 competent cells with cloned plasmids were cultured in 5 mL of LB broth with 100 μL/mL ampicillin for 18 h. Cloned plasmids containing the 16S rDNA were then extracted using a GeneJet Plasmid MiniPrep Kit (ThermoScientific, United States). Sequencing was performed by First BASE Laboratories Sdn Bhd, Singapore, with the use of both 27F and 1492R primers. The 16S rDNA sequences were compared with those available in the NCBI GenBank Database using BlastN at http://blast.ncbi.nlm.nih.gov/Blast.cgi.

### Antimicrobial Resistance Disk Diffusion Test

For each *S. maltophilia* isolate confirmed by the API 20 NE system, the AMR against four different antibiotics (aztreonam, ciprofloxacin, norfloxacin, and sulfamethoxazole-trimethoprim) (Oxoid, Hampshire, United Kingdom) was tested by the disk diffusion method. A bacterial suspension of 0.5 McFarland standard turbidity was first prepared using a 24 h-old culture as described above. A sterile cotton swab was dipped into the bacterial suspension, and the swab was pressed and twisted against the inner surface of the test tube to remove excess fluid. The swab was streaked across a Mueller-Hinton agar (MHA) (Oxoid, Hampshire, United Kingdom) surface in a zigzag manner. The MHA plate was turned 45°clockwise and streaked again using the same swab, and this step was repeated one more time so that the swab had been streaked across the agar a total of three times. The antibiotic disks were placed onto the agar using a pair of sterile forceps. Four antibiotics disks were placed onto the same *S. maltophilia-*inoculated MHA plate, and the plates were incubated at 37°C for 18–20 h. The diameters of the inhibition zones formed were measured, and the resistance status was determined using the Clinical and Laboratory Standards Institute (CLSI) M100 breakpoint values (obtained in November 2018). Due to the lack of CLSI breakpoint values for aztreonam, ciprofloxacin, and norfloxacin for *S. maltophilia*, the breakpoint values for the closed related *Pseudomonas aeruginosa* were used instead. The specific breakpoint values used were: aztreonam (susceptible: ≥ 22 mm; intermediate: 16–21 mm; resistant: ≤15 mm), ciprofloxacin (susceptible: ≥21 mm; intermediate: 16–20 mm; resistant: ≤15 mm), norfloxacin (susceptible: ≥17 mm; intermediate: 13–16 mm; resistant: ≤12 mm), and sulfamethoxazole-trimethoprim (susceptible: ≥16 mm; intermediate: 11–15 mm; resistant: ≤10 mm). *Escherichia coli* (ATCC 25922) was used as the control microorganism.

### Biofilm-Formation Ability Test

For each *S. maltophilia* isolate, a loopful of bacteria was cultured in 10 mL of Tryptone Soya Broth (TSB) (Oxoid, Hampshire, United Kingdom) at 30°C for 24 h. A second round of culturing was performed by transferring 0.1 mL of the TSB cultures into fresh TSB (10 mL) for incubation at 30°C for 24 h. The resultant cultures were diluted 1:100 times using 10% TSB, and 0.2 mL of each of the diluted TSB cultures was transferred into three different wells of a 96-well microtiter plate (Citotest, Nanjing, China). Non-inoculated 10% TSB was used as the negative control. The microtiter plate was incubated at 30°C for 48 h prior to the measurement of biofilm-forming ability. After incubation, TSB was removed from the wells. Each well was then washed with 0.2 mL of Phosphate Buffered Saline (PBS) (Vivantis Technologies Sdn. Bhd., Selangor, Malaysia) thrice in order to remove planktonic bacterial cells prior to the air drying of the microtiter plate in a BSL-2 cabinet for 30 min. The cells were stained with 0.1% crystal violet dye (0.1 mL per well) (Sigma-Aldrich, MO, United States) for 15 min. The dye was removed, and each well was washed with 0.2 mL of deionized water thrice to remove any excess dye. The wells were left to air dry in the BSL-2 cabinet for another 30 min prior to the solubilization of the stained bacterial cells with 33% glacial acetic acid (0.2 mL per well) (VWR Chemicals, PA, United States). The microtiter plate was equilibrated at 4°C to for 15 min. The solution in each well was homogenized by pipetting up and down before transferring 0.125 mL into a new microtiter plate. Absorbance reading was conducted at 595 nm using a microplate photometer (IVD model, Multiskan^TM^ FC, Thermo Fisher Scientific Inc., MA, United States).

The biofilm-forming capacity (measured by the total biofilm biomass) was determined from the criteria established by Stepanović et al. (2007). The optical density cut-off value (OD_c_) is the sum of the average OD of the negative control and three times the standard deviation of the negative control. Biofilm-forming ability is recognized to be “weak” if OD_c_ < OD < 2OD_c_, to be “moderate” if 2OD_c_ < OD < 4OD_c_, and to be “strong” if OD > 4OD_c_.

### Biofilm Treatment With UV and QAC, and Bacterial Enumeration

The procedures for forming biofilm remained the same as in the above description, except that the biofilms were formed in small polystyrene petri dishes (60 × 16 mm, vented, Greiner Bio-One GmbH). For dual-species biofilm, *S. maltophilia* was inoculated into the petri dishes together with *Salmonella enterica* serovar Typhimurium reference strain (ATCC 14028) or self-isolated *Pseudomonas fluorescens* at equal population levels (approximately 10^5^ CFU/petri dish).

For biofilm samples that were exposed to UV treatment, they were first washed with 3 mL PBS to remove planktonic cells and then exposed to UV irradiation. The UV-C dosage applied was measured using a spectrometer (MS-100 Multi-Sense Optical Radiometer, Ultra-Violet Products Ltd., Cambridge, United Kingdom) and confirmed to be 250 mJ/cm^2^ for all of the trials.

Commercial QAC disinfectant (Whisper V, EcolabTM Inc., Saint Paul, MN, United States) was used, containing active ingredients of 3.00% alkyl dimethyl benzyl ammonium chloride, 2.25% octyl decyl dimethyl ammonium chloride, 1.35% didecyl dimethyl ammonium chloride, and 0.90% dioctyl dimethyl ammonium chloride. The disinfectant was diluted with deionized water to 200 ppm according to the recommendation on the product label that this was the concentration suitable for food contact surfaces. The final concentration of 200 ppm was confirmed by QAC colorimetric test strips (Merck, Darmstadt, Germany). Biofilm samples were rinsed with 3 mL PBS to remove loosely attached cells and exposed to 5 mL of disinfectant solution for 15 min. The disinfection solution was then immediately replaced by 5 mL of sterilized D/E neutralizing broth (Acumedia, Lansing, MI, United States) to inactivate the biocidal effects of sanitizers.

The biofilm matrix was recovered by scraping the petri dish surfaces with sterile cell scrapers (SPL Lifescience Co., Ltd., South Korea) for 2 min and serial dilution in peptone salt solution [0.1% peptone (Oxoid), 0.9% Sodium Chloride (Sigma)]. *S. maltophilia*, *S.* Typhimurium, and *P. fluorescens* were enumerated by VIA agar, xylose lysine deoxycholate agar (XLD) (Oxoid), and *Pseudomonas*-CFC supplemented agar (Oxoid), respectively.

### Fate of *S. maltophilia* in Sterile TSB and Basil Juice During a Storage Period of 6 Days at 4 and 20°C

Sweet basil leaves were homogenized together with sterile water at a 1:2 ratio, followed by filtration by stomach bags with a filter to remove large particulate debris. The filtrate was centrifuged at 10,000 rpm for 30 min, and the supernatant was centrifuged again at 12,000 rpm for another 30 min. The resultant supernatant was filter-sterilized through membrane filters (0.22 μm) (Sartorius Stedim Biotech, Göttingen, Germany), and the filtrate was kept frozen at −20°C until use.

*Stenotrophomonas maltophilia* strains [ATCC 13637 (the reference strain) and SM-B1, SM-B3, SM-B4 (self-isolated strains from basil in this study)] were cultured in TSB at 30°C for 24 h for two consecutive passages before being washed with PBS by centrifugation. The bacterial suspensions (0.1 mL, 10^3^–10^4^ CFU/mL) were inoculated into 12-well cell culture cluster plates (Corning Inc., Wujiang, China) and mixed with either TSB or thawed, sterile basil juice to a total volume of 1 mL. The plates were stored at 4 and 20°C for 6 days. Enumeration of *S. maltophilia* was performed on day 0, day 3, and day 6 with VIA agar plates.

### Data Analysis

Statistical analyses were performed via a *T*-test with Microsoft^®^ Office Excel 2016. Significant differences were considered when *P* was <0.05.

## Results

### Isolation and Confirmation of *S. maltophilia*

As shown in Table 1, 11 *S. maltophilia* isolates confirmed by the API 20NE system with variable phenotype profiles and significant taxa percentages were obtained from three types of leafy green vegetables (sweet basil, kale, and parsley) purchased on different dates from a local supermarket and from the farm water (nutrient solution that has direct contact with the roots of plants) of a running hydroponic facility at a local farm. All of the three types of leafy green vegetables (sweet basil, kale, and parsley) were produced by local hydroponic farms according to their packaging.

**TABLE 1:** Profile of *S*. *maltophilia* isolates from leafy green vegetables and hydroponic farming facilities.

**Isolates**	**Source**	**Isolation date**	**API 20 NE profile and significant taxa percentage**	**16S rDNA sequencing confirmed^a^**	**Antibiotics resistance status^b^**				**Biofilm-forming ability^c^**
					**Aztreonam**	**Ciprofloxacin**	**Norfloxacin**	**Sulfamethoxazole-trimethoprim**	
SM-B1	Sweet basil	26/09/2018	0472341, 99.9%	Yes	R	S	S	S	Moderate
SM-B2	Sweet basil	26/09/2018	1472341, 99.9%	Yes	R	S	S	S	Moderate
SM-B3	Sweet basil	08/10/2018	1472341, 99.9%	Yes	R	S	S	S	Strong
SM-B4	Sweet basil	08/10/2018	0472341, 99.9%	Yes	R	S	S	S	Weak
SM-K1	Kale	08/01/2019	0472341, 99.9%		S	S	S	S	Weak
SM-K2	Kale	08/01/2019	0442341, 79.6%	Yes	S	S	S	S	Weak
SM-K3	Kale	08/01/2019	0442341, 79.6%	Yes	I	S	S	S	Moderate
SM-P1	Parsley	08/01/2019	1472341, 99.9%	Yes	R	S	S	S	Strong
SM-F1	Hydroponic farming facility	22/01/2019	0472341, 99.9%	Yes	R	S	S	S	Strong
SM-F2	Hydroponic farming facility	22/01/2019	0472341, 99.9%		R	S	S	S	Weak
SM-F3	Hydroponic farming facility	22/01/2019	0432341, 99.9%		R	S	S	S	Moderate
Reference strain	ATCC13637, clinical strain	NA^d^	NA^d^		S	S	S	S	Strong

*^*a*^Within 99.9% identity with NCBI-documented *S. maltophilia* strains; ^*b*^S, susceptible; I, intermediate; R, resistant; ^*c*^Biofilm-forming capacity determined from criteria established by Stepanović et al. (2007) as described in section “Materials and Methods”; results are based on the average of triplicates; ^*d*^NA, not available.*

**FIGURE 1:**
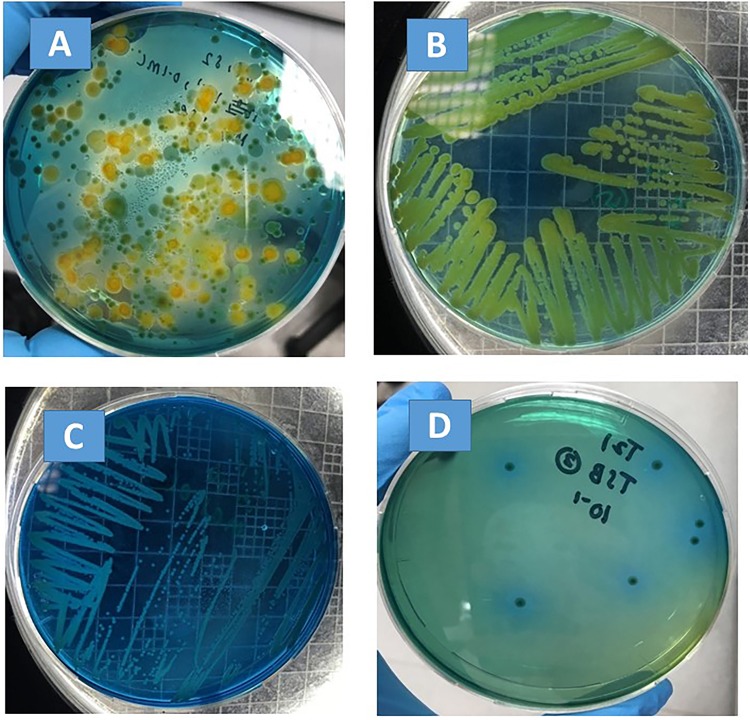
Bacterial morphology on VIA plates. **(A)** Plated with basil extract; **(B)** sub-culture of a green colony from **(A)**, which was confirmed to be *P. fluorescens* by API 20NE; **(C)** sub-culture of a green colony from **(A)**, which was confirmed to be *S. maltophilia* by API 20NE; **(D)** plated with filter-sterilized basil juice inoculated with *S. maltophilia*.

*Stenotrophomonas maltophilia* appears as a dark, translucent, green smear on the VIA agar, and well-isolated colonies are small and circular (Figure 1C). However, when mixed with the background microflora that can also grow on VIA plates (e.g., Figure 1A, plated directly with basil extract), presumptive *S. maltophilia* colonies had to be picked up and sub-cultured on new VIA plates in order to confirm their morphology (e.g., Figures 1B,C were both streaked from green colonies on the plate shown in Figure 1A; the isolate shown in Figure 1B was confirmed to be *P. fluorescens*, and the isolate shown in Figure 1C was confirmed to be *S. maltophilia*).

Eight out of the eleven *S. maltophilia* isolates were further analyzed by 16S rDNA sequencing. All of the eight strains were within 99.9% identity with NCBI-documented *S. maltophilia* strains (Table 1), indicating a rather high 16S rDNA sequence conservativity (Rossi-Tamisier et al., 2015).

### Antimicrobial Resistance of Isolated *S. maltophilia*

The antibiotic resistance of all of the 11 *S. maltophilia* isolates to sulfamethoxazole-trimethoprim, aztreonam, ciprofloxacin, and norfloxacin were measured. Sulfamethoxazole-trimethoprim is the most commonly used antibiotic treatment for *S. maltophilia* infections (Adegoke et al., 2017). In this study, it was observed that all of the 11 *S. maltophilia* isolates from leafy green vegetables and hydroponic farming facilities remained susceptible to sulfamethoxazole-trimethoprim, ciprofloxacin, and norfloxacin. As for aztreonam, 2 out of 11 strains were susceptible, 8 out of 11 strains were resistant, and 1 out of 11 strains was intermediate (Table 1).

### Biofilm-Forming Ability of Isolated *S. maltophilia*

Variable biofilm-forming abilities were observed for the 11 *S. maltophilia* isolates, as determined by the measurement of biomass and from the criteria established by Stepanović et al. (2007) (three strong biofilm formers, four moderate biofilm formers, and four weak biofilm formers, Table 1).

### Susceptibility of *S. maltophilia* Biofilm to UV and QAC Treatment

**FIGURE 2:**
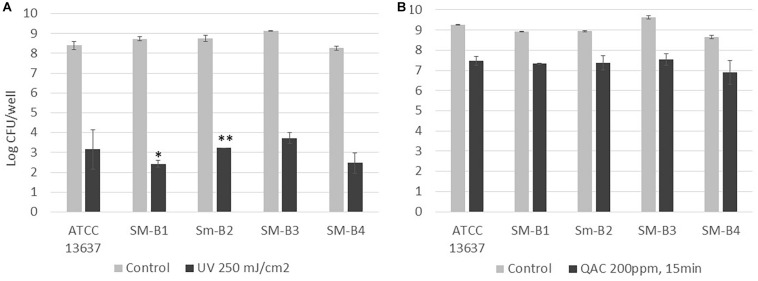
Enumeration of viable cells of *S. maltophilia* biofilms before and after **(A)** UV (250 mJ/cm^2^) and **(B)** QAC (200 ppm for 15 min) treatment. Each column represents the average of triplicates, and each error bar indicates the standard error. ^∗^One of the triplicates was below the 1.7 log CFU/well detection limit; ^∗∗^two of the triplicates were below the 1.7 log CFU/well detection limit.

UV and QAC are two representative antimicrobial treatments that can be applied by hydroponic farms to increase microbial safety. The viable *S. maltophilia* cells enumerated from biofilms of the reference strain (ATCC 13637) and the four strains isolated from sweet basil (SM-B1, SM-B2, SM-B3, SM-B4) were comparable, at 8 to 9-log CFU/well (Figures 2A,B). For all of the tested *S. maltophilia* strains in biofilms, 250 mJ/cm^2^ UV treatment induced higher reductions (5 to 6-log reductions, Figure 2A) than a QAC treatment of 200 ppm for 15 min (1 to 2-log reductions, Figure 2B). Therefore, the former treatment (250 mJ/cm^2^ UV) was selected for the following tests to compare between single-species and dual-species biofilms mixed with a known strong biofilm-former, *S.* Typhimurium ATCC 14028 (Vestby et al., 2009; De Oliveira et al., 2014), as well as *P. fluorescens*, which was co-isolated with *S. maltophilia* from basil in this study (Figure 1B).

Three independent trials (biological replicates) were performed to compare single-species and dual-species biofilms of *S. maltophilia* ATCC 13637 mixed with *S.* Typhimurium ATCC 14028 and *P. fluorescens*, respectively (Table 2, each data point represents the average of triplicates [technical replicates]). Before UV treatment, no significant difference was observed in the population levels of *S. maltophilia* in single-species biofilms and in dual-species biofilms (*p* > 0.05). After UV treatment, when *S. maltophilia* was mixed with *S.* Typhimurium ATCC 14028, in two out of the three trials, lower population levels of *S. maltophilia* were observed in the dual-species biofilms (<1.7 log CFU/well) than in single-species biofilms (3.5 ± 0.2 and 2.8 ± 0.4 log CFU/well) (Table 2). On the contrary, when *S. maltophilia* was mixed with *P. fluorescens*, in two out of the three trials, higher population levels of *S. maltophilia* were observed in dual-species biofilms (2.6 ± 0.7 and 3.6 ± 0.3 log CFU/well) than in single-species biofilms (<1.7 log CFU/well) (Table 2).

**TABLE 2:** Enumeration of viable cells (Log CFU/well ± standard error) of *S. maltophilia* (ATCC 13637) in single-species and dual-species biofilms mixed with **(A)** *S.* Typhimurium (ATCC 14028) and **(B)** *P. fluorescens* (self-isolated from basil).

**A**								

**Independent**	***S. maltophilia* in**		***S. maltophilia* in**		***S.* Typhimurium in**		***S.* Typhimurium in**	
**trial number**	**single-species biofilms**		**dual-species biofilms**		**single-species biofilms**		**dual-species biofilms**	
				
	**Control**	**UV treated**	**Control**	**UV treated**	**Control**	**UV treated**	**Control**	**UV treated**

1	>8.2^a^	3.5 ± 0.2	>8.2^a^	<1.7^b^	>8.2^a^	<1.7^b^	>8.2^a^	<1.7^b^
2	7.8 ± 1.1	2.8 ± 0.4	9.0 ± 0.3	<1.7^b^	7.9 ± 0.1	<1.7^b^	8.1 ± 0.1	<1.7^b^
3	9.0 ± 0.2	<1.7^b^	9.6 ± 0.2	<1.7^b^	9.2 ± 0.1	<1.7^b^	9.0 ± 0.1	<1.7^b^

**B**								

**Independent**	***S. maltophilia* in**		***S. maltophilia* in**		***P. fluorescens* in**		***P. fluorescens* in**	
**trial number**	**single-species biofilms**		**dual-species biofilms**		**single-species biofilms**		**dual-species biofilms**	
				
	**Control**	**UV treated**	**Control**	**UV treated**	**Control**	**UV treated**	**Control**	**UV treated**
1	8.4 ± 0.0	2.0^c^	8.4 ± 0.0	2.0^c^	8.2 ± 0.2	1.7^c^	8.3 ± 0.3	<1.7^b^
2	8.0 ± 0.2	1.7^c^	7.9 ± 0.1	2.6 ± 0.7	8.1 ± 0.1	1.7^c^	8.3 ± 0.1	3.0 ± 1.1^d^
3	8.6 ± 0.0	<1.7^b^	7.8 ± 0.2	3.6 ± 0.3	8.2 ± 0.1	<1.7^b^	8.3 ± 0.1	2.6 ± 0.7

*Each data point represents the average of triplicates. ^*a*^All plates were too many to count, >300 colonies per plate (TNTC), corresponding to >8.2 log CFU/well; ^*b*^below detection limit, <1 colony per plate, corresponding to <1.7 log CFU/well; ^*c*^data from one of the triplicates, the other two replicates were below the detection limit; ^*d*^data from the average of two out of the triplicates, the other replicate was below the detection limit.*

### Fate of *S. maltophilia* in Sterile TSB and Basil Juice During a Storage Period of 6 Days at 4 and 20°C

The fate of *S. maltophilia* during a storage period of 6 days at 4 and 20°C in filter-sterilized TSB and basil juice was tested. No colony was observed for the negative controls (VIA plates plated with the non-inoculated basil juice). Colonies with consistent morphology were observed on VIA plates that were plated with inoculated basil juice (e.g., Figure 1D). At 4°C, *S. maltophilia* was able to survive, but no growth was observed for any of the tested strains (ATCC 13637, SM-B1, SM-B3, SM-B4) in both TSB and basil juice during a storage period of 6 days (Table 3). At 20°C, all the tested strains (ATCC 13637, SM-B1, SM-B3, SM-B4) were able to grow substantially (an over 5-log increase) in both TSB and basil juice within 3 days.

**TABLE 3:** Fate of *S. maltophilia* in sterile TSB and basil juice during a storage period of 6 days at 4 and 20°C.

**Temperature**	**Strain**	**Enumeration of *S. maltophilia* (log CFU/mL ± SD)**					
		
		**In TSB**			**In basil juice**		
		**Day 0**	**Day 3**	**Day 6**	**Day 0**	**Day 3**	**Day 6**
4°C	ATCC 13637	4.0 ± 0.3	3.5 ± 0.0	3.5 ± 0.1	4.0 ± 0.3	3.8 ± 0.4	3.6 ± 0.2
	SM-B1	2.9 ± 0.1	2.5 ± 0.2	2.9 ± 0.1	2.9 ± 0.1	2.5 ± 0.2	2.8 ± 0.1
	SM-B3	3.2 ± 0.0	3.0 ± 0.2	3.0 ± 0.1	3.2 ± 0.0	3.0 ± 0.1	3.1 ± 0.0
	SM-B4	3.0 ± 0.0	3.0 ± 0.0	3.0 ± 0.1	3.0 ± 0.0	3.0 ± 0.0	3.0 ± 0.2
20°C	ATCC 13637	3.3 ± 0.1	>8.8^a^	NT^b^	3.3 ± 0.1	>8.8^a^	NT^b^
	SM-B1	2.9 ± 0.1	>8.8^a^	NT^b^	2.9 ± 0.1	>8.8^a^	NT^b^
	SM-B3	3.2 ± 0.0	>8.8^a^	NT^b^	3.2 ± 0.0	>8.8^a^	NT^b^
	SM-B4	3.0 ± 0.0	>8.8^a^	NT^b^	3.0 ± 0.0	>8.8^a^	NT^b^

*Each data point represents the average of triplicates. ^*a*^All plates were too many to count (TNTC). Calculation was based on plate surface area (58 cm^2^) and highest dilution factor (10^–5^); ^*b*^NT, not tested.*

## Discussion

*Stenotrophomonas maltophilia* is not only an important nosocomial pathogen but also an environmental globally emerging multiple-drug-resistant microorganism, which has previously been found both inside and outside of hospital/clinical settings, including on plants and animals and in water treatment and distribution systems (Brooke, 2012). According to the report of Qureshi et al. (2005), *S. maltophilia* was cultured from 14 (78%) of 18 salads. In this study, *S. maltophilia* was isolated from all of the leafy green vegetables (sweet basil, kale, and parsley) and farm water samples tested. Due to the limited differentiation ability of VIA agar between *S. maltophilia* and background microflora, a secondary purification had to be done in order to confirm the bacterial morphology. As a result, 38 presumptive colonies were picked out in total from the three types of leafy green vegetables and the hydroponic farm water, and 16 sub-cultures were produced, with API 20 NE confirmation. Twelve out of the 16 sub-cultures were confirmed to be *S. maltophilia.* Table 1 shows that 11 out of the 12 confirmed isolates showed different API 20 NE profiles, antibiotics resistance statuses, and/or biofilm-forming abilities, indicating that they are different strains with genetic variabilities. Eight out of the 11 isolates were further analyzed by 16S rDNA sequencing, and all of them were shown to be within 99.9% identity with NCBI-documented *S. maltophilia* strains.

Although it was not possible to perform an accurate enumeration of *S. maltophilia* from the leafy green vegetable and farm water samples based on the current culture-based method, the 11 *S. maltophilia* isolates were obtained from various dilutions of the vegetable extracts from 10^–1^ to 10^–4^, indicating that the population levels of *S. maltophilia* on leafy green vegetables can be as high as up to 10^5^ CFU/g. In fact, much effort was expended in this study to determine the survival/growth potentials of *S. maltophilia* on leafy green vegetables. A durability test was carried out to follow up on the fate of the innate *S. maltophilia* on basil tissues, and a challenge test was performed to follow up on the fate of the spiked *S. maltophilia* reference strain on basil tissues during storage at 4 and 20°C. Unfortunately, both tests were severely affected by the microflora background. Therefore, in the end, the fate of *S. maltophilia* was tested in filter-sterilized TSB and basil juice as a simulated leafy green vegetable environment, in which *S. maltophilia* was able to maintain a stable population level at 4°C during a storage period of 6 days, and was able to grow by over 5-log within 3 days. Lastly, a constant high level of viable cells (8 to 9-log CFU/well) was detected from the biofilms formed by all of the four *S. maltophilia* isolates, which showed variable biofilm-forming abilities on the basis of the measurement of biomass (one weak biofilm former, two moderate biofilm formers, one strong biofilm former). *S. maltophilia* is not a highly virulent pathogen. However, the presence of high concentrations of *S. maltophilia* in foods or food-contact surfaces does pose a potential risk of infection due to dose-response relationships (Brouwer et al., 2017). *S. maltophilia* has been reported to be able to acquire DNA from environmental bacteria (Brooke, 2012). Although the *S. maltophilia* isolated in this study remained susceptible to most of the tested antibiotics, including sulfamethoxazole-trimethoprim, which is the most commonly used antibiotic treatment for *S. maltophilia* infections, high population levels of *S. maltophilia*, especially mixed with other bacteria in biofilms, have a risk for horizontal gene transfer which may generate new multiple drug-resistant strains.

Leafy green vegetables are nutritious and are well recognized as healthy foods. Many leafy green vegetables, including the three types tested in this study (sweet basil, kale, and parsley), are, in general, consumed raw or with minimal cooking procedures. However, since leafy green vegetables naturally harbor a high surface population of microbes and are often associated with microbial pathogen transmission, it is not recommended for the aging population or vulnerable individuals facing immunosuppressive diseases to consume leafy green vegetables without proper cooking (Berg et al., 2014). Consumers tend to perceive urban farming, especially hydroponic produced leafy green vegetables, as “clean products” since there is usually no soil involved, and they are a lot less prone to contamination from animals. However, as shown in this study, high levels of opportunistic pathogens can still be present on hydroponically produced leafy green vegetables, very probably due to the nutrient solution (farm water) used in the hydroponic farming facilities.

Currently, variable farming practices are used at different urban agriculture facilities. This study investigated the possibilities of applying UV and/or chemical sanitizers in order to enhance the microbial safety of hydroponic farms. Doses of UV (250 mJ/cm^2^) and QAC (200 ppm, 15 min) used in this study were recognized as harsh treatments by previous literature (Li et al., 2014; Pang et al., 2019). This study showed that 250 mJ/cm^2^ UV treatment induced higher reductions (5 to 6-log reduction) than a QAC treatment of 200 ppm for 15 min (1 to 2-log reduction) for all of the tested *S. maltophilia* strains in biofilms, indicating that it is a more effective and feasible strategy for adoption by hydroponic farms to treat their nutrient solutions that come into contact with the plants and any relevant surfaces.

In the natural environment, biofilms are usually formed by multiple bacterial species, and mixed-species biofilms are reported to show increased resistance to multiple treatments in comparison with single-species biofilms (Modic et al., 2017; Pang et al., 2019). Therefore, in this study, in order to investigate whether the inactivating effect of UV could be attenuated by the presence of other bacteria species, the UV-resistance of *S. maltophilia* was not only tested in single-species biofilms but also in dual-species biofilms. Surprisingly, when mixed with *S.* Typhimurium ATCC 14028, *S. maltophilia* in dual-species biofilms was reduced to an even lower level than in single-species biofilms by the same dose of UV treatment, suggesting a possible antagonistic effect between the tested *S. maltophilia* and *S.* Typhimurium strains. On the other hand, when *P. fluorescens* co-isolated from basil was mixed with *S. maltophilia* in this study, the UV resistance of *S. maltophilia* was indeed increased in comparison with single-species biofilms. Nevertheless, it must be noticed that substantial reductions (>4-log reductions) of *S. maltophilia* were able to be achieved by 250 mJ/cm^2^ UV in all of the tested scenarios.

All in all, this study demonstrated the common presence of an emerging opportunistic pathogen *S. maltophilia* in leafy green vegetables produced by hydroponic farms as well as in the nutrient solutions used by a hydroponic farm. In a simulated leafy green vegetable environment (sterile basil juice), all of the tested strains showed an ability to maintain their viability at 4°C and great growth potential at 20°C, indicating the importance of avoiding temperature abuse in the food chain. Although with variable biofilm-forming abilities as determined by biomass measurement, which might be due to different secretion levels of extracellular polymeric substances (EPS), all of the tested *S. maltophilia* strains showed comparably high populations in biofilms on plastic materials, indicating the important role that biofilms play in the associated transmission of *S. maltophilia*. Lastly, it was recommended that UV treatment be adopted by hydroponic farms to mitigate possible microbial risks.

## Data Availability Statement

The raw data supporting the conclusions of this manuscript will be made available by the authors, without undue reservation, to any qualified researcher.

## Author Contributions

DL is the PI who planned the work and drafted the manuscript. MS and NK are final year undergraduate students who finished their thesis under the guidance of DL on this topic and done most of the lab work. CW provided excellent technical work and performed the additional tests during the manuscript revision.

## Conflict of Interest

The authors declare that the research was conducted in the absence of any commercial or financial relationships that could be construed as a potential conflict of interest.
